# Integrating *Systems Biology Markup Language* (SBML) with NEURON

**DOI:** 10.1186/1471-2202-15-S1-P144

**Published:** 2014-07-21

**Authors:** Anna S Bulanova, Robert A McDougal, Samuel A Neymotin, Victor K Mutai, William W Lytton, Michael L Hines

**Affiliations:** 1 Neurobiology, Yale University, New Haven, CT 06520, USA; 2 Physiology & Pharmacology, SUNY Downstate Medical Center, Brooklyn, NY 11203, USA; 3 Neurology, Kings County Hospital, Brooklyn, NY 11203, USA

## 

The NEURON simulator software is widely used by the computational neuroscience community for neuronal electrophysiology modeling and we have recently extended it to support reaction-diffusion dynamics [1]. This class of dynamics is ideal for studying intracellular dynamics, such as calcium-induced calcium release. NEURON’s original reaction-diffusion support introduced a custom syntax; although this allowed taking advantage of NEURON’s full capabilities, it required existing cell biology models to be rewritten. To eliminate this barrier, we have introduced SBML support for NEURON.

SBML [2] is an XML-based representation format used for specifying computational models of biological processes. This format is one of the most widely used, and is supported by over 200 software packages. It has become a standard used by systems biologists for the exchange of information about chemical reaction dynamics. We have introduced SBML support to the reaction diffusion module of the NEURON simulation software. This enables NEURON users to import a large number of previously developed cell biology models and use them in computational neuroscience research.

We have added the capability to import and export models in SBML format to NEURON’s reaction-diffusion module using the libSBML library [3]. This allows one to combine models from ModelDB [4] with cell models from the BioModels database [5] in a way that makes it possible to match state variables and to readily change parameters. The procedure is as follows:

• Electrophysiology model is loaded from ModelDB or constructed de novo.

• NEURON loads SBML data and instantiates appropriate reaction diffusion objects: rxd.Region, rxd.Species, rxd.Reaction. User must interactively or preemptively match state-variable names across combined models to combine the two models into one.

• SBML models do not include diffusion so diffusion constants can be added to provide the spatial element of the combined model.

• User can adjust the parameters and make simulation runs of the model.

We have used these new features to study the interaction of a calcium induced calcium release model from BioModels with electrical activity in a detailed model of a hippocampal CA1 pyramidal neuron.
